# Paternal race/ethnicity and risk of adverse birth outcomes in the United States, 1989–2013

**DOI:** 10.3934/publichealth.2018.3.312

**Published:** 2018-08-16

**Authors:** Yu Li, Zhehui Luo, Claudia Holzman, Hui Liu, Claire E. Margerison

**Affiliations:** 1 Department of Epidemiology, Brown University, USA; 2 Department of Epidemiology and Biostatistics, Michigan State University, USA; 3 Department of Sociology, Michigan State University, USA

**Keywords:** preterm birth, paternal race/ethnicity, small for gestational age, mixed-race couple, adverse birth outcomes

## Abstract

**Objectives:**

Investigate adverse birth outcomes in the United States (US) from 1989–2013 in relation to paternal and maternal race/ethnicity.

**Design:**

We used US natality data for singleton births to women 15–44 with information on birthweight, gestational age, and covariates (n = 90,771,339). We calculated unadjusted and adjusted probabilities of preterm birth (PTB, < 37 weeks gestation) and small for gestational age (SGA, < 10^th^ percentile) among all combinations of maternal and paternal race/ethnicity: non-Hispanic black (NHB), non-Hispanic white (NHW), Hispanic, and Asian, and where paternal race/ethnicity was missing.

**Results:**

Missing, followed by NHB, paternal race/ethnicity had the two highest risks of PTB within each maternal racial/ethnic group. Asian, followed by NHW, paternal race/ethnicity had the two lowest risks of PTB. For SGA, however, Asian, followed by missing, paternal race/ethnicity had the two highest risks, and NHW race/ethnicity had the lowest risk. Our findings also demonstrate effect modification on the additive scale, with missing and NHB paternal race/ethnicity conferring a larger increase in risk of PTB for NHB women compared to women of other race/ethnicity groups.

**Conclusions:**

These data confirm US disparities in adverse birth outcomes by maternal and paternal race/ethnicity and argue for increased resources and interventions in response.

## 1. Introduction

Non-Hispanic black (NHB) women have had a higher risk of delivering infants either too small or too early compared to Hispanic women and non-Hispanic white (NHW) women for as long as data have been collected in the United States (US) [1]. In 2014, for example, black women were almost twice as likely to deliver preterm compared to Asian women, Hispanic women, and white women (13.0% vs. 8.5%, 9.0% and 8.9%) [1]. Proposed explanations for these racial disparities in birth outcomes include racial/ethnic differences in socioeconomic status (SES), age at pregnancy, marital status, quality of health care, genetic factors, neighborhood of residence, and life time exposure to stress and discrimination [2]–[4]. Yet, none of these factors, nor any combinations, fully explain why birth outcomes differ so substantially and persistently by race in the US [5].

Much less research has examined the contribution of paternal race to birth outcomes. The increasing prevalence of mixed-race couples in the US makes it increasingly important to understand whether and how paternal race/ethnicity contributes uniquely to birth outcomes, above and beyond maternal race/ethnicity. Growing evidence suggests that couples with one NHB partner have worse birth outcomes than couples where both partners are NHW [6]–[8]. Indeed, a meta-analysis of 8 studies found higher odds of low birthweight (LBW, OR: 1.2 and 1.8), preterm birth (PTB, OR: 1.2 and 1.4), and stillbirth (OR: 1.4 and 1.5) among both white mother/black father and black mother/white father couples compared to white mother/white father couples [9].

One important gap in the existing literature is that few studies have examined maternal/paternal race/ethnicity combinations for racial/ethnic groups other than NHB and NHW [8]. The only study to include other racial/ethnic groups used data from New York City 2000–2010 and found that all mixed-race couples had higher risk of adverse birth outcomes (LBW, small for gestational age [SGA], PTB, and infant mortality) when compared to NHW/NHW couples. However, these findings may not generalize to the rest of the US due to the unique nature of the New York City population in terms of race/ethnicity, country of origin of minority racial/ethnic groups (e.g., many Hispanic mothers are of Puerto Rican or Dominican origin), and SES.

Another important gap is that most previous studies have excluded births with “missing” paternal race/ethnicity, yet “missingness” of paternal race/ethnicity may be an important risk factor. Infants whose paternal race/ethnicity was unreported on their birth certificates have higher risk of infant mortality and morbidity compared to infants with the same maternal race/ethnicity where paternal race/ethnicity is not missing [10],[11]. Although the reasons for “missing” paternal race/ethnicity data on birth records are often unknown, missingness may suggest a lack of paternal involvement, which has been shown to be associated with adverse birth outcomes [12].

Moreover, to our knowledge, there has been no examination of the contribution of paternal race/ethnicity to adverse birth outcomes using US national birth data more recent than 2001 [13]. Our study, therefore, sought to address these gaps by assessing the contribution of paternal race to two important adverse birth outcomes, PTB and SGA, using US national birth data from 1989 to 2013. We examined the four most prevalent maternal and paternal racial/ethnic groups, and included “missing” paternal race/ethnicity as a separate category. We hypothesized that, within each maternal racial/ethnic group, (1) the lowest risk of PTB or SGA would be found when paternal race/ethnicity was NHW; and (2) the highest risk of PTB or SGA would be found when paternal race/ethnicity was “missing”.

## 2. Materials and Methods

### 2.1. Data and study population

Our study population included singleton births to US resident women age 15 to 44 years old in the natality file from 1989 to 2013 (n = 97,903,276). The analytic sample included records from the selected study population with available data on maternal age, race/ethnicity, nativity, marital status, education, parity, birthweight and gestational age (n = 93,299,604). We applied criteria from Alexander et al. (2003) to eliminate implausible birthweights [15] (remaining n = 91,987,377). Records with either maternal or paternal American Indian/Alaska Native or multiple race/ethnicities as well as births in US territories were excluded for a total analytic sample of 90,771,339 singleton births. Due to small sample sizes, we did not include American Indian/Alaska Natives or those reporting multiple race/ethnicities.

### 2.2. Measures

Our primary dependent variables were: (1) PTB (< 37 weeks gestation), and (2) SGA, defined as less than 10th percentile of weight for gestational age based on published standards for all racial/ethnic groups combined [15]. We used the National Center for Health Statistics' “best estimate” of gestational age, which primarily uses gestational age based on date of last menstrual period (LMP) but in a small percentage (∼5% or less) of cases where LMP is missing or incompatible with birthweight, gestational age is imputed based on a clinical/obstetric estimate.

Our main explanatory variables were maternal and paternal race/ethnicity, based on the race and ethnicity information listed on the birth certificate. Those whose ethnicity belonged to Mexican, Puerto Rican, Cuban, Central or South American, or “Other and unknown Hispanic” were defined as Hispanic, regardless of their race. Individuals not identifying as Hispanic were then categorized as non-Hispanic white (NHW), non-Hispanic black (NHB), or Asian/Pacific Islander (hereafter, “Asian”, which included Chinese, Japanese, Filipino, Asian Indians, and “Other Asian/Pacific Islander”). Those with paternal race/ethnicity blank or listed as “missing” were categorized as “Missing”.

We selected covariates likely associated with both maternal/paternal race/ethnicity as well as adverse birth outcomes: parity (number of previous live births and categorized as 0 [reference], 1, or ≥ 2), marital status (married [reference] vs unmarried), maternal education level (less than high school, high school, college, more than college [reference]), nativity (US born [reference], foreign born) and maternal age ( < 20, 20–29 [reference], 30–39, ≥ 40). These covariates may confound the association between race/ethnicity and adverse birth outcomes; however, because these variant may also mediate the association between race/ethnicity and birth outcomes, we also present both unadjusted and adjusted estimates and compare the two. We did not control for smoking and access of prenatal care because these are most likely to be potential mediators and not confounders.

### 2.3. Statistical analysis

We first calculated the unadjusted probability of both PTB and SGA within each maternal/paternal race/ethnicity group. We hypothesized that, within each maternal race/ethnic group, the lowest risk of both outcomes would be among infants with NHW paternal race/ethnicity. To test this hypothesis, we compared the unadjusted risk difference between each paternal race/ethnic group and the NHW group, stratified by maternal race/ethnicity.

We then used logistic regressions to estimate the adjusted relationship between maternal/paternal race/ethnicity combinations and PTB and SGA. We adjusted these models for year of birth and state of birth to control for secular trends and time-invariant state characteristics that could be associated with the probabilities of both mixed-race/ethnicity couples as well as of PTB and SGA. We also adjusted for individual-level covariates: maternal age, parity, maternal nativity, maternal education, and marital status. From the logistic regression models, we then estimated the predicted probability of PTB and SGA for each maternal/paternal race/ethnicity category, holding the covariates constant at their reference categories.

## 3. Results

First, characteristics of the study population are presented in Table 1. In our population of over 90 million singleton US births, almost 59% were to NHW mothers, 15% were to NHB mothers, 21% were to Hispanic mothers, and about 5% were to Asian mothers. The most common maternal/paternal race/ethnicity combinations were NHW/NHW at 50%, Hispanic/Hispanic at 15%, and NHB/NHB at 9% (Column 2, Table 1). Infants born to NHB mothers were most likely to have missing paternal race/ethnicity (37.96%), followed by Hispanic mothers (13.20%), NHW mothers (9.11%), and Asian mothers (5.13%) (Column 3, Table 1). About 41% of all births were to nulliparous mothers, 66% were to married mothers, and 79% were to native born mothers. Approximately one-third of mothers had a high school education or GED, 23% had some college education, and 15% had a college diploma. The majority of births were to mothers between the ages of 20 and 39. Table S1 of Supplementary gives details on demographic characteristics by parental race/ethnicity groups.

**Table 1.** Characteristics of the study sample, singleton births to US resident women age 15 to 44 years old from 1989 to 2013(n = 90,771,339 births).

Race/ethnicity			Total Frequency		% of total sample		% within maternal race/ethnic group		
Maternal	Paternal								
Non-Hispanic white (NHW)									
	NHW		45,559,015		50.19		84.30		
	NHB		1,113,311		1.23		2.06		
	Hispanic		2,056,974		2.27		3.81		
	Asian		390,507		0.43		0.72		
	Missing		4,920,997		5.42		9.11		
	All		54,040,804		59.54				
Non-Hispanic black (NHB)									
	NHW		303,238		0.33		2.22		
	NHB		7,934,869		8.74		58.19		
	Hispanic		195,816		0.22		1.44		
	Asian		26,220		0.03		0.19		
	Missing		5,175,807		5.70		37.96		
	All		13,635,950		15.02				
Hispanic									
	NHW		1,727,674		1.90		9.24		
	NHB		448,153		0.49		2.40		
	Hispanic		13,937,222		15.35		74.5		
	Asian		124,951		0.14		0.67		
	Missing		2,469,375		2.72		1		
	All		18,707,375		20.61				
Asian									
	NHW		698,565		0.77		15.92		
	NHB		100,210		0.11		2.28		
	Hispanic		145,153		0.16		3.31		
	Asian		3,218,248		3.55		73.36		
	Missing		225,034		0.25		5.13		
	All		4,387,210		4.83				
Parity									
Nulliparous			37,135,151		40.91				
Primaparous			29,284,946		32.26				
Multiparous			24,351,242		26.83				
Marital status									
Married			59,621,905		65.68				
Unmarried			31,149,434		34.32				
Education attainment									
0–8 years			5,133,797		5.66				
9–11 years			14,121,856		15.56				
HS grad/GED			28,462,106		31.36				
Some college			21,049,080		23.19				
College grad			13,980,080		15.40				
More than college			8,024,420		8.84				
Nativity									
Native born			71,472,566		78.74				
Foreign born			19,298,773		21.26				
Age									
< 20			9,994,192		11.01				
20–29			48,366,027		53.28				
30–39			30,522,619		33.63				
≥ 40			1,888,501		2.08				

Table 2 shows the unadjusted probabilities of PTB and SGA among all maternal/paternal racial/ethnic combinations. NHB mothers had the highest risk of PTB at 0.16 for all paternal race/ethnicities combined (compared to 0.08, 0.10, and 0.09 for NHW, Hispanic, and Asian mothers, respectively). Within each maternal race/ethnicity group, the highest probability of PTB was found for infants with missing paternal race/ethnicity; risks ranged from 0.12 for NHW mothers to 0.18 for NHB mothers. Contrary to our hypothesis, within each maternal race/ethnicity group, the lowest risk of PTB was for infants with Asian paternal race/ethnicity. The unadjusted risk differences demonstrate the difference in risk conferred by pairing with a father of a race/ethnic group other than NHW (which we hypothesized would be lowest), within each maternal race/ethnicity group. The risk conferred by having missing paternal race/ethnicity is highest for NHB mothers (0.06), indicating that among NHB mothers, 6 out of 100 additional infants are born preterm when paternal race/ethnicity is missing compared to when paternal race/ethnicity is NHW. On the other hand, risk conferred by pairing with an NHB father is lowest for NHW mothers, for whom the additional risk compared to pairing with an NHW father is just 0.02 compared to 0.03 for every other maternal race/ethnic group.

The unadjusted findings in Table 2 tell a different story for SGA compared to PTB. For SGA, NHB and Asian mothers have higher overall risks at 0.14 and 0.13, respectively, compared to NHW and Hispanic mothers. Within each maternal race/ethnicity group, Asian and missing paternal race/ethnicity have the two highest risks of SGA. The risk conferred by missing paternal race/ethnicity is higher in both NHB and Asian mothers at 0.05 compared to 0.04 in NHW and Hispanic mothers. The risk conferred by pairing with an Asian father is highest among Asian mothers at 0.05 compared to 0.02, 0.03, and 0.03 for NHW, NHB, and Hispanic mothers with Asian fathers, respectively. Table S2 of Supplementary shows results when only state and year of birth are included in the model; findings are similar to those in Table 2.

Table 3 shows the adjusted probabilities of PTB and SGA by maternal/paternal race/ethnicity group along with 95% confidence intervals from multivariable logistic regression models. Again, we see that the highest probabilities of PTB tend to appear among NHB mothers. However, after adjustment for maternal characteristics, the risk of PTB associated with missing and NHB paternal race/ethnicity is lower than in the unadjusted analyses (for all maternal race/ethnicities), and for NHW and NHB mothers, the association between Hispanic paternal race/ethnicity and PTB is now equivalent to the association for NHB fathers. Asian paternal race/ethnicity is still associated with the lowest probability of PTB within each maternal race/ethnicity group. Overall, the highest risk of PTB is found among NHB mothers with missing paternal race/ethnicity (0.11, 95% CI: 0.09, 0.14), followed closely by NHB mothers and Asian mothers paired with NHB fathers (0.10, 95% CI: 0.07, 0.14; and 0.10, 95% CI: 0.08, 0.13, respectively).

The adjusted models for SGA tend to demonstrate the highest probabilities for NHB mothers (for each paternal race/ethnic group), followed by Asian mothers. Within each maternal race/ethnicity group, the risk of SGA is highest for Asian fathers followed by the missing paternal race/ethnicity group. Overall, the highest risk of SGA is found among Asian/Asian maternal/paternal race/ethnicity combinations (0.15, 95% CI: 0.11, 0.24), followed by NHB mothers paired with Asian fathers (0.13, 95% CI: 0.10, 0.21).

**Table 2.** Frequencies and unadjusted probabilities and risk differences (RD) for preterm birth (PTB) and small for gestational age (SGA) by maternal and paternal race/ethnicity among singleton births in the US from 1989 to 2013(n = 90,771,339 births).

Race/ethnicity		PTB			SGA		
Maternal	Paternal	Frequency	Probability	RD	Frequency	Probability	RD
NHW	NHW	3,600,464	0.08	Reference	3,264,868	0.07	Reference
	NHB	109,959	0.10	0.02	103,645	0.09	0.02
	Hispanic	179,537	0.09	0.01	179,250	0.09	0.02
	Asian	27,816	0.07	-0.01	37,045	0.10	0.02
	Missing	572,432	0.12	0.04	572,397	0.12	0.04
	All	4,490,208	0.08		4,157,205	0.08	
							
NHB	NHW	36,223	0.12	Reference	32,923	0.11	Reference
	NHB	1,146,331	0.15	0.03	1,052,825	0.13	0.02
	Hispanic	25,250	0.13	0.01	25,807	0.13	0.02
	Asian	2,975	0.11	-0.01	3,632	0.14	0.03
	Missing	919,148	0.18	0.06	805,081	0.16	0.05
	All	2,129,927	0.16		1,920,268	0.14	
							
Hispanic	NHW	154,124	0.09	Reference	130,921	0.08	Reference
	NHB	50,881	0.11	0.03	45,844	0.10	0.03
	Hispanic	1,369,283	0.10	0.01	1,259,355	0.09	0.01
	Asian	11,002	0.09	0.00	13,386	0.11	0.03
	Missing	309,408	0.13	0.04	275,819	0.11	0.04
	All	1,894,698	0.10		1,725,325	0.09	
							
Asian	NHW	64,872	0.09	Reference	56,955	0.08	Reference
	NHB	12,199	0.12	0.03	10,275	0.10	0.02
	Hispanic	16,274	0.11	0.02	15,370	0.11	0.02
	Asian	267,177	0.08	-0.01	437,928	0.14	0.05
	Missing	29,647	0.13	0.04	30,380	0.14	0.05
	All	390,169	0.09		550,908	0.13	

**Table 3.** Multivariate adjusted^1^ predicted probabilities and 95% confidence intervals (95% CI) for preterm birth and small for gestational age, by maternal and paternal race/ethnicity.

Race/ethnicity		PTB		SGA	
Maternal	Paternal	Predicted probability	95% CI	Predicted probability	95% CI
NHW	NHW	0.06	0.04, 0.09	0.07	0.05, 0.13
	NHB	0.07	0.05, 0.10	0.08	0.06, 0.14
	Hispanic	0.07	0.05, 0.09	0.08	0.06, 0.14
	Asian	0.06	0.04, 0.08	0.10	0.07, 0.16
	Missing	0.07	0.05, 0.10	0.08	0.06, 0.15
					
NHB	NHW	0.09	0.07, 0.12	0.10	0.07, 0.17
	NHB	0.10	0.07, 0.14	0.12	0.09, 0.20
	Hispanic	0.10	0.08, 0.12	0.12	0.08, 0.18
	Asian	0.09	0.07, 0.11	0.13	0.10, 0.21
	Missing	0.11	0.09, 0.14	0.12	0.09, 0.17
					
Hispanic	NHW	0.07	0.05, 0.10	0.07	0.05, 0.13
	NHB	0.08	0.07, 0.11	0.09	0.06, 0.15
	Hispanic	0.07	0.05, 0.10	0.08	0.06, 0.14
	Asian	0.07	0.06, 0.10	0.10	0.08, 0.17
	Missing	0.08	0.07, 0.10	0.08	0.06, 0.14
					
Asian	NHW	0.08	0.06, 0.12	0.09	0.07, 0.15
	NHB	0.10	0.08, 0.13	0.10	0.07, 0.17
	Hispanic	0.09	0.08, 0.12	0.11	0.08, 0.17
	Asian	0.08	0.06, 0.11	0.15	0.11, 0.24
	Missing	0.10	0.08, 0.12	0.11	0.08, 0.19

^1^Note: Adjusted model includes fixed effect covariates (birth year, state) as well as maternal age, nativity, education, parity and marital status.

## 4. Comments

Using US vital statistics natality data for all singleton births from 1989 to 2013, our study investigated the contribution of paternal race/ethnicity to two important adverse birth outcomes, PTB and SGA. As hypothesized, we found that, within each maternal race/ethnicity group, “missing” paternal race/ethnicity was associated with the highest risk of PTB, followed by NHB paternal race/ethnicity. Contrary to our hypothesis, Asian, not NHW, paternal race/ethnicity was associated with the lowest risk of PTB. For SGA, on the other hand, missing, followed by Asian, paternal race/ethnicity was associated with the highest risks within each maternal race/ethnicity group.

Our findings also showed that, for the maternal racial/ethnic group with the highest overall risk of each outcome (i.e., NHB for PTB and Asian for SGA), pairing with a father of the same racial/ethnic group, or having missing paternal race/ethnicity, conferred a higher risk than other pairings for maternal race/ethnic groups. For example, for NHB mothers, pairing with NHB or missing paternal race/ethnicity was associated with an excess of 3 to 6 preterm births out of 100 live births compared to pairing with a NHW father, whereas for a NHW mother, these excess values were only 2 and 4, respectively. This implies effect modification on the additive scale, where the association between missing and NHB paternal race/ethnicity and PTB is stronger for NHB mothers compared to the associations for mothers of other racial/ethnic groups.

Our finding that infants with NHB fathers had higher risks of both PTB and SGA compared to those with NHW fathers regardless of mother's race/ethnicity was consistent with previous studies [7],[13],[16],[17]. Our study also included two racial/ethnic groups not previously examined in national studies of paternal race/ethnicity: Hispanic and Asian. The only previous study including these two race/ethnicity groups used data from New York City. A notable difference is that this previous study found elevated relative risk of both PTB and SGA for all mothers (except white mothers) pairing with Asian fathers, whereas we found that Asian paternal race/ethnicity was protective against PTB (but not SGA), compared to all other paternal race/ethnic groups. Moreover, the previous study also found that Hispanic paternal race/ethnicity conferred similar or even higher risk of PTB than black paternal race/ethnicity for white, black, and Asian mothers. Reasons that our findings differed from this study likely include the different countries of origin and socioeconomic position of the Hispanic and Asian populations in New York City compared to the entire US, and possibly the fact that the previous study only included urban populations.

Our findings that Asian maternal and paternal race/ethnicity was consistently associated with increased risk of SGA (compared to other racial/ethnic groups) but that Asian maternal and paternal race/ethnicity had the lowest risks of PTB indicated that Asian infants may not be pathologically unhealthy but may simply have a smaller body size. A previous study from Canada also showed that if parental race/ethnicity was Asian, infant birthweight was lower than white European parental race/ethnicity [18]–[20]. Thus, separate growth standards may be appropriate for births to either Asian mothers or fathers.

Within all maternal race/ethnicity groups, the risk of both PTB and SGA was highest for records where paternal race/ethnicity was missing, which is consistent with previous studies [21]–[23]. Missing paternal race/ethnicity may be a marker for other characteristics [10],[11]. For example, prior research has indicated that birth records with missing paternal information are also more likely to indicate that the mother have inadequate prenatal care or smoked during pregnancy [11],[23],[24]. Our data (not shown, available from the authors upon request) confirmed these results.

Our findings imply both biological and social impacts of paternal race/ethnicity for adverse birth outcomes. For example, the fact that Asian paternal race/ethnicity is associated with higher risk of SGA across maternal race/ethnicity groups likely suggests a biological mechanism whereby infants of Asian descent are smaller in size. This is further supported by the fact that adjustment for key social variables such as education, marital status, age, and parity does not substantially alter the probability of SGA in the Asian paternal race/ethnicity categories. On the other hand, the association between NHB paternal race/ethnicity and PTB changes more substantially in adjusted estimates. This suggests that mothers pairing with a NHB father differ on social and demographic factors compared to NHW mothers pairing with other race/ethnicity groups. The remaining (after adjustment) difference in risk of PTB associated with NHB paternal race/ethnicity compared to NHW paternal race/ethnicity may be due to factors similar to those hypothesized to explain the black-white disparity in PTB by maternal race, e.g., neighborhood, discrimination, or chronic stress [25]. Future studies should investigate the mechanisms that explain the association between paternal race/ethnicity and adverse birth outcomes.

There were several important limitations in our study. We were not able to adjust for father's socioeconomic status or level of involvement in the pregnancy, limiting our ability to investigate mechanisms behind the associations identified. For example, father's education was only recorded from 1989 to 1995, and from 2010 to 2013. In addition, all data, including both maternal and paternal race/ethnicity, maternal education, maternal age, parity, marital status and nativity were self-reported. Moreover, those with missing paternal race/ethnicity could technically be considered misclassified, as they should technically be in one of the other race/ethnicity categories; this misclassification could result in biased estimates for the groups from which they are missing. However, we believe that the “missing” category does represent a different role of fathers in the pregnancy. Finally, there was potential measurement error in gestational age because it was primarily based on LMP [15], and LMP is not always accurate enough as reported by women themselves [26].

A main strength of this study was that it used a very large sample across 25 years throughout the whole US, which represented almost all singleton births from 1989 to 2013 and reduced concerns about the external validity of the results. The availability of data on paternal race/ethnicity was more than 80% in each year. Most previous studies of race/ethnicity-combination couples and birth outcomes only included white-black couples. Interracial couples including Hispanics and Asians had not been included except for one study [8]. Previous studies only accounted for a short period of time or included only a few states.

There are growing numbers of families with different parental race/ethnicity combinations due to increased immigration [27] and greater acceptance of interracial dating and marriage. Only 48% of the American public supported whites dating blacks in 1987, while this percentage had increased to 83% in 2009 [28]; thus we must begin to gain a better understanding of birth outcomes among these families. Our findings also suggest that more paternal information, such as information on education, and socioeconomic status, should be included on birth certificates to increase our understanding of the mechanisms behind observed paternal race/ethnicity disparities in birth outcomes. These data, which provide further evidence of the racial disparities in adverse birth outcomes, may be used to inform resource allocation within public health and suggest the need to test public health policies and interventions to reduce these disparities.
