# Supplementary material for: Paternal race/ethnicity and risk of adverse birth outcomes in the United States, 1989–2013
Source: AIMS Public Health. 2018 Aug 16;5(3):312–23. doi: 10.3934/publichealth.2018.3.312 (PMC6141552; doi:10.3934/publichealth.2018.3.312)
Supplement: Supplementary file 1 [file publichealth-05-03-312-s001.pdf]

AIMS Public Health

AIMS Public Health, 5(3): 312–323.
DOI: 10.3934/publichealth.2018.3.312
Received: 11 June 2018
Accepted: 10 August 2018
Published: 16 August 2018

http://www.aimspress.com/journal/aimsph

Research article

Paternal race/ethnicity and risk of adverse birth outcomes in the United States, 1989–2013

Yu Li[1], Zhehui Luo[2], Claudia Holzman[2], Hui Liu[3] and Claire E. Margerison[2,*]

[1] Department of Epidemiology, Brown University, USA
[2] Department of Epidemiology and Biostatistics, Michigan State University, USA
[3] Department of Sociology, Michigan State University, USA

* **Correspondence:** Email: margeris@msu.edu; Tel: +5173538623; Fax: +5173538623.

Table S1. Demographic characteristics by maternal/paternal race/ethnicity combination groups.

Maternal characteristics	Maternal/Paternal race/ethnicity combination[1]																			
	WW	WB	WH	WA	WM	BW	BB	BH	BA	BM	HW	HB	HH	HA	HM	AW	AB	AH	AA	AM
	Column Percent																			
Age																				
< 20	6.0	13.9	12.6	5.2	27.1	10.5	13.4	17.2	10.4	28.4	7.5	16.7	13.5	10.8	27.0	2.1	8.1	10.0	2.5	16.6
20–29	51.4	60.4	56.6	45.1	56.4	54.2	56.1	58.5	54.1	56.4	50.8	60.4	57.6	54.1	54.7	34.5	52.1	50.4	43.7	53.6
30–39	40.3	24.1	29.0	46.3	15.2	32.5	28.5	22.8	32.7	14.3	39.1	21.7	27.1	33.1	17.0	58.2	36.7	36.9	50.7	26.9
≥ 40	2.3	1.6	1.8	3.5	1.3	2.7	2.0	1.5	2.8	1.0	2.7	1.3	1.8	2.1	1.3	5.3	3.0	2.8	3.1	2.8
Education																				
0–8 years	1.4	1.4	2.0	0.7	4.1	0.9	1.7	1.3	0.9	3.0	2.2	1.9	22.8	2.2	20.3	1.0	1.2	1.3	5.2	11.2
9–11 years	8.0	17.2	16.0	5.8	30.4	11.0	15.8	17.6	9.9	34.3	10.4	20.3	27.5	12.8	37.6	3.1	8.5	9.7	6.6	21.8
HS grad	29.5	37.7	34.2	22.6	40.6	30.9	37.9	35.6	30.1	41.3	30.6	37.6	29.2	35.5	28.9	17.8	32.6	30.2	21.1	36.2
Some college	26.2	28.2	27.0	26.0	19.4	33.0	28.6	31.9	34.1	18.1	30.2	29.5	14.0	29.2	10.9	24.0	32.3	30.5	18.7	19.0
College grad	22.4	9.9	13.0	24.9	3.6	14.8	10.5	9.0	15.3	2.5	16.6	7.3	4.2	12.7	1.6	31.3	17.3	18.9	27.1	7.9
More than college	12.5	5.5	7.8	20.1	1.9	9.5	5.5	4.6	9.7	0.9	10.0	3.5	2.2	7.6	0.7	22.7	8.2	9.5	21.3	3.9
Nativity																				
Native born	94.5	95.1	94.1	89.9	96.6	85.5	86.2	89.3	78.3	94.5	69.0	77.0	33.0	69.7	47.7	30.6	34.7	47.2	10.7	27.1
Foreign born	5.5	5.0	5.9	10.2	3.4	14.5	13.8	10.7	21.7	5.5	31.0	23.0	67.0	30.3	52.3	69.4	65.3	52.8	89.4	72.9
Marital status																				
Married	85.5	43.3	65.9	82.8	8.3	61.0	47.2	42.4	60.4	2.4	76.5	39.6	60.7	65.3	5.4	90.1	62.8	65.8	91.2	12.5
Unmarried	14.5	56.7	34.1	17.2	91.6	39.1	52.8	57.6	39.7	97.6	23.5	60.4	39.3	34.7	94.7	9.9	37.2	34.2	8.8	87.5
Parity																				
Nalliparous	41.2	42.3	42.3	45.2	54.0	44.3	36.8	43.7	44.0	41.1	43.4	42.4	34.2	42.8	45.6	49.5	44.7	47.1	45.3	52.7
Primaparous	34.7	30.8	31.6	32.9	24.4	30.9	30.8	29.2	31.0	26.8	33.2	29.7	30.8	31.8	25.0	33.7	30.8	31.1	35.7	25.1
Multiparous	24.1	26.9	26.1	21.9	21.6	24.8	32.5	27.1	25.0	32.1	23.4	27.9	35.0	25.4	29.4	16.8	24.5	21.8	19.0	22.3

[1]Note: In column headers, the first letter is for maternal race/ethnicity and the second letter is for paternal race/ethnicity. W=white; B=black; H=Hispanic; A=Asian and M=missing.

Table S2. Multivariate adjusted[1] predicted probabilities and 95% confidence intervals (95% CI) for preterm birth and small for gestational age, by maternal and paternal race/ethnicity.

		PTB		SGA	
Maternal race/ethnicity	Paternal race/ethnicity	Predicted probability	95% CI	Predicted probability	95% CI
NHW	NHW	0.08	0.05, 0.11	0.07	0.05, 0.12
	NHB	0.10	0.06, 0.13	0.09	0.07, 0.15
	Hispanic	0.09	0.05, 0.12	0.09	0.06, 0.14
	Asian	0.07	0.04, 0.10	0.10	0.07, 0.16
	Missing	0.11	0.07, 0.16	0.12	0.09, 0.19
NHB	NHW	0.12	0.07, 0.16	0.11	0.08, 0.18
	NHB	0.14	0.09, 0.19	0.13	0.10, 0.21
	Hispanic	0.13	0.08, 0.18	0.13	0.10, 0.21
	Asian	0.12	0.07, 0.16	0.14	0.10, 0.22
	Missing	0.17	0.11, 0.23	0.16	0.11, 0.25
Hispanic	NHW	0.09	0.05, 0.13	0.08	0.05, 0.13
	NHB	0.12	0.07, 0.16	0.10	0.07, 0.17
	Hispanic	0.10	0.06, 0.14	0.09	0.07, 0.15
	Asian	0.09	0.05, 0.13	0.11	0.08, 0.18
	Missing	0.12	0.08, 0.17	0.11	0.08, 0.18
Asian	NHW	0.10	0.06, 0.13	0.08	0.06, 0.14
	NHB	0.12	0.08, 0.17	0.11	0.08, 0.17
	Hispanic	0.12	0.07, 0.16	0.11	0.08, 0.18
	Asian	0.09	0.05, 0.12	0.14	0.10, 0.22
	Missing	0.14	0.08, 0.18	0.14	0.10, 0.22

[1]Note: Adjusted model includes fixed effect covariates (birth year, state).

AIMS Press

© 2018 the Authors, licensee AIMS Press. This is an open access article distributed under the terms of the Creative Commons Attribution License (http://creativecommons.org/licenses/by/4.0)
